# Lipid Exchange Mechanism of the Cholesteryl Ester Transfer Protein Clarified by Atomistic and Coarse-grained Simulations

**DOI:** 10.1371/journal.pcbi.1002299

**Published:** 2012-01-12

**Authors:** Artturi Koivuniemi, Timo Vuorela, Petri T. Kovanen, Ilpo Vattulainen, Marja T. Hyvönen

**Affiliations:** 1 Department of Physics, Tampere University of Technology, Tampere, Finland; 2 Wihuri Research Institute, Helsinki, Finland; 3 Department of Applied Physics, Aalto University School of Science and Technology, Espoo, Finland; 4 MEMPHYS – Centre for Biomembrane Physics, University of Southern Denmark, Odense, Denmark; 5 Department of Physics, University of Oulu, Oulu, Finland; Fox Chase Cancer Center, United States of America

## Abstract

Cholesteryl ester transfer protein (CETP) transports cholesteryl esters, triglycerides, and phospholipids between different lipoprotein fractions in blood plasma. The inhibition of CETP has been shown to be a sound strategy to prevent and treat the development of coronary heart disease. We employed molecular dynamics simulations to unravel the mechanisms associated with the CETP-mediated lipid exchange. To this end we used both atomistic and coarse-grained models whose results were consistent with each other. We found CETP to bind to the surface of high density lipoprotein (HDL) -like lipid droplets through its charged and tryptophan residues. Upon binding, CETP rapidly (in about 10 ns) induced the formation of a small hydrophobic patch to the phospholipid surface of the droplet, opening a route from the core of the lipid droplet to the binding pocket of CETP. This was followed by a conformational change of helix X of CETP to an open state, in which we found the accessibility of cholesteryl esters to the C-terminal tunnel opening of CETP to increase. Furthermore, in the absence of helix X, cholesteryl esters rapidly diffused into CETP through the C-terminal opening. The results provide compelling evidence that helix X acts as a lid which conducts lipid exchange by alternating the open and closed states. The findings have potential for the design of novel molecular agents to inhibit the activity of CETP.

## Author Summary

Coronary heart disease is a major cause of death in the Western societies. One of the most promising interventions to prevent and slow down the progress of coronary heart disease is the elevation of high density lipoprotein (HDL) levels in circulation. Animal models together with early clinical studies have shown that the inhibition of cholesteryl ester transfer protein (CETP) is a promising strategy to achieve higher HDL levels. However, drugs with acceptable side-effects for CETP-inhibition do not yet exist, although the next generation CETP inhibitor (anacetrapib) has great potential in this regard. In this study, our objective is to gain more detailed information regarding the interactions of CETP with lipoprotein particles. We show how the CETP-lipoprotein complex is formed and how lipid exchange between CETP and lipoprotein particles takes place. Our findings help to understand in a mechanistic way how CETP-mediated lipid exchange occurs and how it could be exploited in the design of new and more efficient molecular agents against coronary heart disease.

## Introduction

Cholesteryl ester transfer protein (CETP) is a 476-residue-long glycoprotein which promotes the transfer of cholesteryl esters (CEs), triacylglycerols (TGs) and phospholipids (PLs) between the different lipoprotein fractions (high density lipoprotein (HDL), low density lipoprotein (LDL), and very low density lipoprotein (VLDL)) in human blood plasma. CETP is believed to mediate the transfer by a hetero-exchange mechanism in which CEs are carried from HDL to VLDL and LDL particles, and TGs are carried in the opposite direction from VLDL and LDL to HDL particles, resulting in CE depletion and TG enrichment of HDL [1]. Interestingly, CETP is structurally homologous to the phospholipid transfer protein (PLTP), the lipopolysaccharide binding protein (LBP), and the bactericidal/permeability-increasing protein (BPI) [1]. As all these proteins are able to bind phospholipids, similarity in their transportation mechanisms has been suggested. Importantly, however, CETP is the only protein able to transfer neutral lipids (cholesteryl esters and triglycerides) in human plasma [2].

The broad interest to understand CETP and its lipid trafficking properties stems from the fact that it has a potentially protective role in the development of cardiovascular diseases, in particular atherosclerosis, which are currently the main cause of death in Western countries, claiming ∼17 million lives a year. The role of CETP in the development of atherosclerosis became evident when it was found that CETP deficiency and the inhibition of CETP lower LDL and increase HDL levels in human plasma [3]. High HDL levels have been clinically found to be inversely correlated with the development of atherosclerosis, since HDL particles are considered crucial components in the transport of cholesterol from atherosclerotic plaques back into the systemic circulation. Unfortunately, the clinical trial with the first oral anti-atherogenic drug candidate with a CETP-inhibitory activity, torcetrapib, was unsuccessful because of its potentially lethal side effects [4]. Treatment with torcetrapib increased blood pressure and circulating aldosterone levels and also altered serum electrolyte levels. However, subsequent studies indicated that these adverse effects of torcetrapib were unrelated to the inhibition of CETP and are not necessarily shared by the other members of the class of CETP inhibitors. Indeed, a recent clinical trial showed that another CETP inhibitor, anacetrapib, effectively raises HDL and has an acceptable side-effect profile in patients with coronary heart disease or risk factors for coronary heart disease [5]. Importantly, a recent meta-analysis of 92 studies involving 113,833 participants concluded that the CETP genotypes that have lower CETP activity are associated with a decreased coronary risk [6].

**Figure 1:**
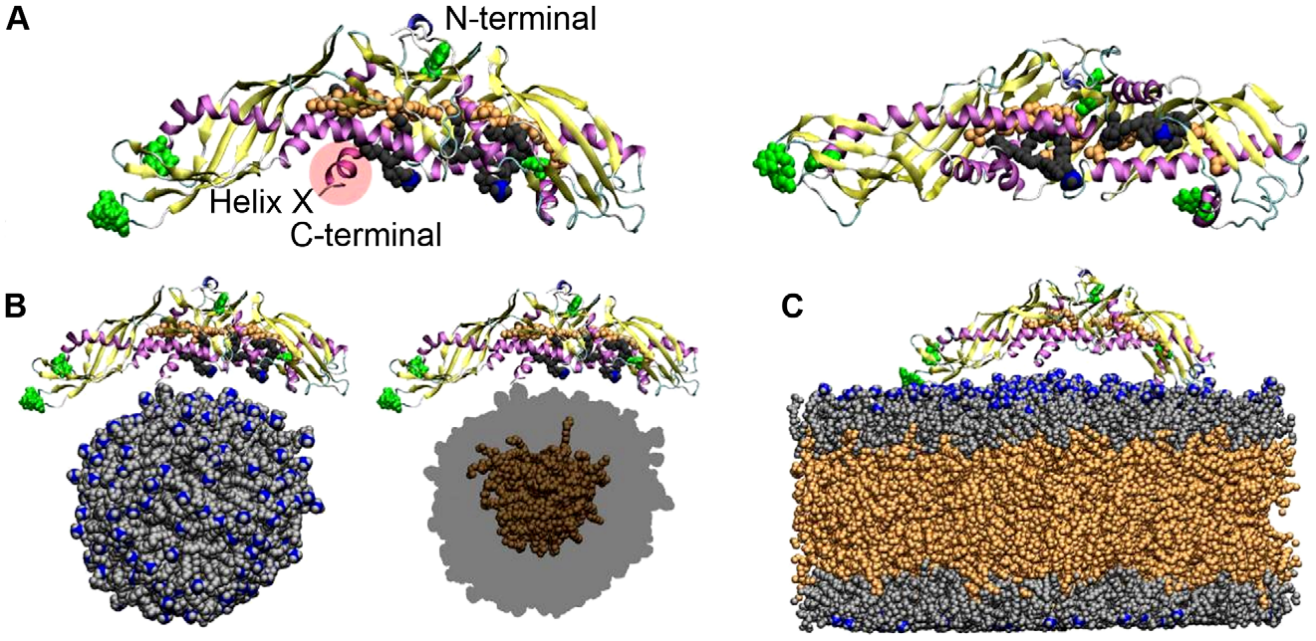
Structure of CETP and starting configurations for simulations. A) X-ray structure of CETP from the side (left) and bottom (right). Two DOPCs (grey and blue spheres) plug the tunnel openings that lead to the hydrophobic tunnel where two CETP-bound CEs (orange spheres) are located. Helix X is labelled and marked with a red sphere. B) The starting configuration for droplet simulations. C) The starting configuration for lipid trilayer simulation. POPCs and DOPCs are coloured as grey, CEs are orange, head group nitrogens are blue, and Trp residues green. Water molecules were removed from the snapshots for clarity.

Considering the central role of CETP in the development of coronary atherosclerosis and its complications, we face an outstanding challenge to better understand the mechanisms associated with CETP functions. Recently, Qiu et al. resolved the X-ray structure of CETP showing that it carries CE molecules inside a long hydrophobic tunnel, whose ends are plugged by phospholipids (Figure 1) [7]. This kind of hydrophobic tunnel is unique among proteins, and it was speculated that CEs diffuse into and out from the tunnel through the two tunnel openings, which are closed by PLs during the transportation in aqueous surroundings. In addition, based on the X-ray structure it has been speculated that CETP is attached to lipoproteins via its concave surface where also the two hydrophobic tunnel openings reside [7]. Further, it has been proposed that the formation of CETP-lipoprotein complexes is modulated by pH, surface pressure, and the ionic interactions between CETP and phospholipids [8], [9]. Fluorescence quenching has been used to demonstrate that the interaction between tryptophan residues of CETP and PLs could be important in the attachment [10]. Regarding the lipid exchange mechanism of CETP, helix X has been suggested to play a role in lipid loading and unloading by acting as a lid at the C-terminal tunnel opening, being in the open state when the exchange of lipids takes place, and in the closed state when CETP detaches from the lipoprotein surface to become surrounded by aqueous medium [5]. Various mutational studies further suggest that helix X is possibly crucial in the transfer of CEs and TGs but not in the transfer of PLs [11], [12].

The above findings and suggestions are appealing and insightful, but call for better understanding of the structure-function relationship and of the dynamics that drive CE, TG and PL transfer. In essence, atomic and molecular scale insight into the lipid exchange between CETP and lipoproteins is limited, which largely stems from exceptional difficulties to experimentally probe the related transient processes in the nanometer scale. In the current study, our objective is to complement experiments through atomistic and coarse-grained molecular dynamics simulations to investigate the binding of CETP to a small lipid droplet and a planar lipid trilayer, and to determine the initial stages of the lipid exchange mechanism. By doing so, we can follow the lipid exchange in atomic detail, shed light on its mechanism, consider the effect of lipoprotein curvature, and unravel the dynamics of the related processes. These mechanisms and phenomena are considered over a multitude of time scales by bridging atomistic and coarse-grained simulations, which are shown to provide consistent results. The present study paves the way for future simulations to elucidate interactions of anacetrapib with CETP and CETP-lipoprotein complexes, with an objective to unlock its inhibitory mechanism. Given the significant role of CETP in cardiovascular diseases, the broad interest of the topic is hoped to attract substantial interest to extend the present work.

## Results

### Flexible structure of CETP helps it to bind to curved lipoproteins

We carried out three 100 ns atomistic simulations for fully hydrated systems containing CETP with different interior lipid compositions and a small pre-equilibrated HDL-sized lipid droplet composed of POPCs and CEs (A1, A2, A3; Figure 1; see Materials and Methods). In addition to these spherical droplets, CETP was simulated with a pre-equilibrated planar POPC-CE trilayer system (A4; Figure 1C) to study the effect of less curved lipoprotein particles, like VLDL and LDL, on the conformation of CETP.

**Figure 2:**
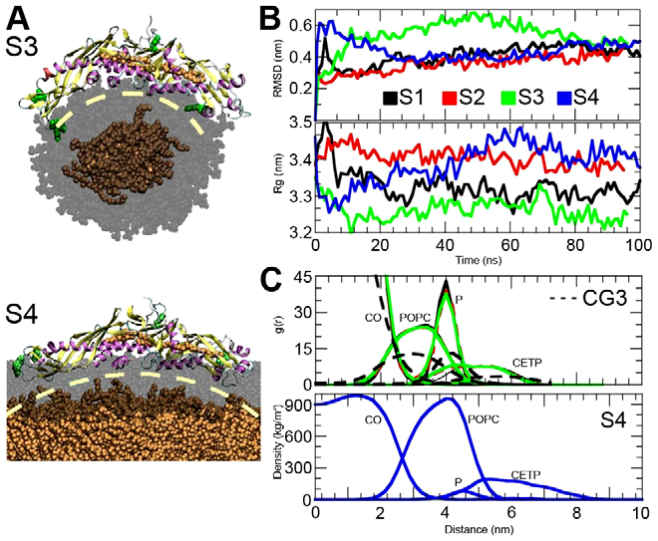
The binding of CETP to lipid surfaces with different curvatures. A) Snapshots from the end of atomistic simulations A3 and A4. POPCs are transparent and grey, and COs are orange. Water molecules were omitted for clarity. CETP is rendered using secondary structures and Trp residues are marked with green color. Dashed and yellow lines present the curvature of CETP. B) RMSD and radii of gyration profiles for CETP in droplet and trilayer simulations. C) Radial distribution functions and density profiles for the droplet and trilayer systems, respectively.

Root mean square deviation (RMSD) profiles indicate that the structures do not deviate considerably from the X-ray structure (Figure 2B). The radius of gyration fluctuated between 3.2 and 3.5 nm, and its profiles together with snapshots from simulation trajectories show that the conformation of CETP is able to bend to bind to surfaces with different curvatures (Figure 2A). In the case of spherical A3 the curvature of CETP is clearly higher than in the planar A4 system, and it became apparent that the conformation of CETP is not able to rearrange sufficiently to fully match the planar surface. Nonetheless, our results imply that the structure of CETP is elastic and facilitates the binding of CETP to different lipoprotein surfaces with varying curvatures. Yet, due to its inherent curvature that closely matches the curvature of HDL, CETP prefers to bind to HDL-sized particles compared to larger VLDL-sized particles. Consequently, we propose that the free energy change associated with the binding of CETP to HDL is more favorable compared to the formation of a CETP-VLDL complex.

Radial distribution functions and density profiles indicate that CETP does not penetrate deeper than to the level of POPC phosphate groups (Figure 2C). Therefore, in all atomistic simulations the core CEs were observed not to interact directly with CETP, as instead they were found to reside only in the core. This suggests that the surface-core lipid ratio is important for the exchange of neutral lipids by CETP.

During the simulation A2, CETP-bound DOPCs did not diffuse into the lipid droplet. However, we found that during the simulation S1 the hydrophobic tunnel of CETP collapsed, which strongly suggests that the structure of CETP is not stable without interior lipids (See Figure S2 and Text S2). This finding is important regarding the lipid exchange process of CETP as it suggests that during the neutral lipid exchange, the hydrophobic cavity is not empty at any point. We return to this matter later.

### Salt bridges and tryptophans stabilize CETP-lipoprotein complexes

**Figure 3:**
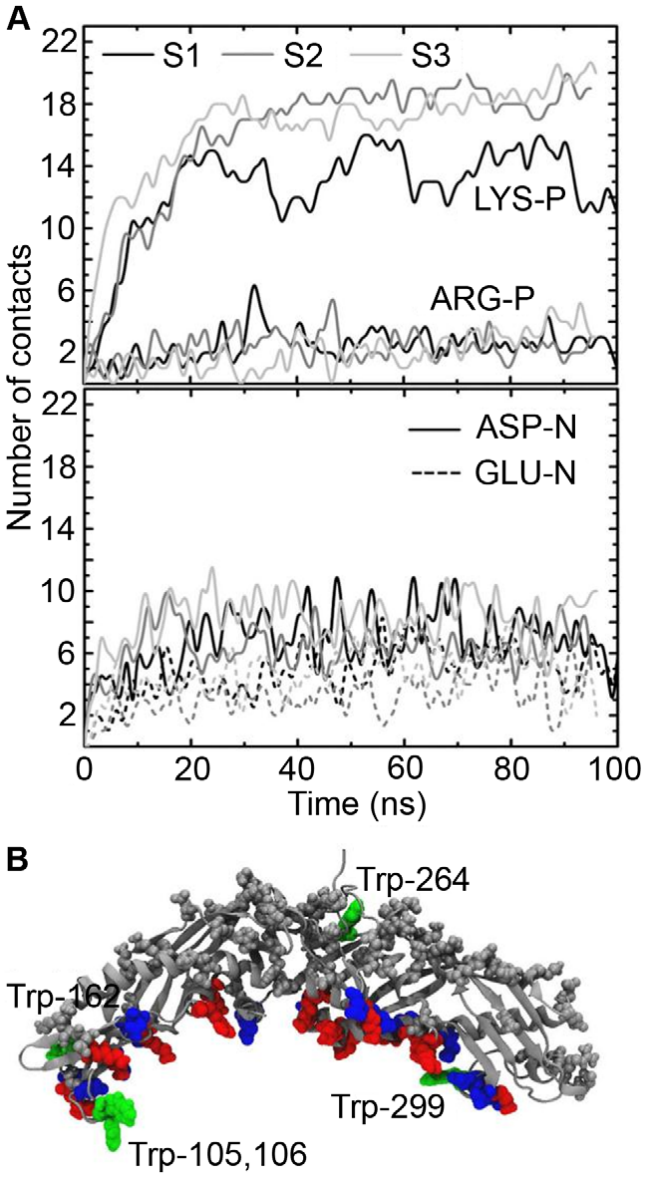
Electrostatic interactions between CETP and lipid droplet. A) Number of salt bridges formed between the charged residues of CETP and the head groups of POPCs as a function of time. The upper profile shows the number of contacts between the positively charged residues and P atoms of POPCs, and the lower profile shows the number of contacts between the negatively charged amino acids and N atoms of POPCs. B) Salt bridge-forming positively (red) and negatively (blue) charged amino acids marked to the structure of CETP. Trp residues are labeled and green.

In atomistic lipid droplet simulations, we calculated the number of salt bridges that formed between CETP and POPCs as a function of time, in order to characterize the key charged residues involved in the attachment of CETP. The number of salt bridges that formed between the positively charged lysine residues of CETP and the negatively charged phosphate (P) groups of POPCs stabilized to a level of 12–20 (Figure 3A). Salt bridging of lysines is much more efficient in A2 and A3 than in A1 (19–20 compared to 12, see Figure 3A). The number of salt-bridges between arginines and P groups was on average two or three. Additionally, we calculated the number of salt bridges formed by the negatively charged Asp and Glu residues and found that Asp residues were able to form 6–8 and Glu residues 2–4 salt bridges with the positively charged choline groups. Amino acids that form most of the salt bridges are shown in Figure 3, revealing that they are mainly located at the edge of the concave surface of CETP.

In the spirit of the earlier Trp quenching study [10], we inspected more carefully the behavior of Trps during binding. In all droplet simulations, Trp299 formed hydrogen bonds with POPCs (Figure 3B). Trp264 stayed buried inside the structure of the protein and Trp162 was able to interact with the water molecules. In A1 and A2, Trp105 and Trp106 were located facing the water phase, while in A3 the flap Ω_5_ interacted with POPCs by anchoring Trps 105 and 106 to the carbonyl region of POPC surface, highlighted in Figure 2. In the trilayer simulation only two Trp residues (105 and 299) were able to interact with the POPC surface.

Our results highlight the importance of electrostatic interactions between CETP and phospholipids in the formation of CETP-droplet complexes. The results provide compelling evidence that three Trp residues anchor CETP to lipid droplets, introducing additional stability to CETP-lipoprotein complexes where the curvature of CETP and a lipoprotein matches.

### Coarse-grained simulations reveal that the ratio of surface and core lipids in lipid droplets is an important modulator of CETP activity

Interpretation of atomistic simulations requires care due to the limited time and length scales that are feasible through atomistic studies. For example, the diffusion of lipids in HDLs is slow compared to the time scales we have simulated and, thus, claims regarding the principal binding site and penetration depth of CETP must be carefully considered. In order to add liability to our atomistic simulations, we also carried out coarse-grained simulations, covering time scales beyond 2 µs.

CG simulations support and validate atomistic simulations by showing that the concave surface is the principal lipoprotein binding site of CETP. We did not observe any deviations from this conclusion during the three independent 2-microsecond simulations. Radial distribution functions shown in Figure 2 depict a similar distribution of molecules as in atomistic simulations.

**Figure 4:**
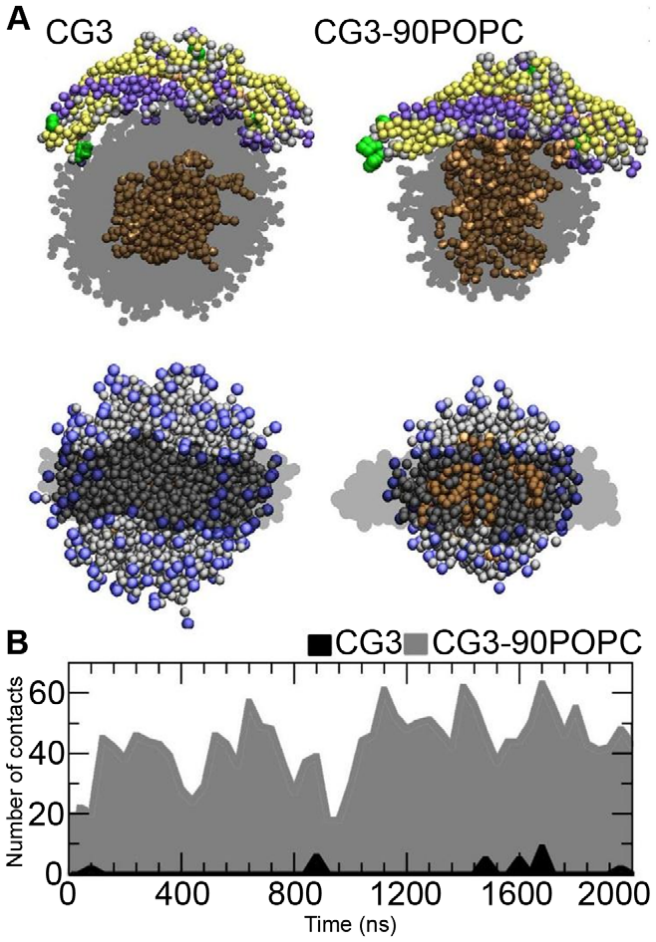
Interaction of CETP with core lipids. A) Snapshots from the coarse-grained simulations CG3 (left) and CG3-90POPC (right). The upper snapshots show side views and the lower ones top views of CETP bound to a lipid droplet. In the latter, the hydrophobic patch under CETP is clearly visible. The structure of CETP has been rendered using secondary structure information in upper snapshots (β-sheets are yellow, α-helixes violet and random coils gray) or as dark transparent phantom in lower snapshots. The green spheres are Trp residues. POPCs are transparent in the upper snapshots, while those in the bottom snapshots are visible as grey (the choline head groups are visible as blue). CEs are rendered with orange spheres. Water molecules were omitted for clarity. B) Number of contacts between core CEs and CETP with different surface-core lipid ratios as a function of time.

However, intriguingly we found that POPCs which were in contact with the concave surface of CETP migrated away from the tunnel openings, forming a small hydrophobic patch under the concave surface (Figure 4A). In essence, CETP drives phospholipids to diffuse away from the slightly hydrophobic tunnel openings to its edges where most of the salt bridge-forming amino acids reside. We analyzed the spatial densities of the polar beads (GL1, GL2, and NC3, PO4, using the descriptions of the Martini model) of POPCs to clarify the patch formation more clearly. The spatial density map revealed the formation of a hydrophobic patch under the concave surface, and specifically in the region where the N- and C-terminal tunnel openings reside (see Figure S1 and Text S1 in Supporting Information (SI)).

The time associated with the formation of the hydrophobic patch is difficult to estimate accurately, since the process fluctuates depending on the dynamics of the CETP-droplet complex. In practice we found the patch to emerge in roughly 10–40 ns, and it increased to a size of about 1 nm×3 nm in 100–500 nanoseconds, depending on the system studied (see Figure S1). At longer times the patch fluctuated quite a lot but there was a trend showing a slow increase in size, suggesting that the total formation time may be of the order of microseconds.

To gain further support for patch formation, as predicted by CG simulations, we repeated the analysis with two additional CG simulations where we used PME for electrostatics with the non-polarizable Martini water model, and PME with the polarizable Martini water model [13]. With the polarizable water model the solubility of charged species to apolar media should be better described compared to the standard Martini model. In both additional CG simulations, hydrophobic patch formation was observed too (Figure S1).

Given that the patch emerged in CG simulations in tens of nanoseconds, and the atomistic simulations lasted for 100 ns, we returned to our atomistic simulation data to consider this aspect in atomic detail. We analyzed the spatial density profile of POPC head groups and observed similar hydrophobic patch formation. For example, in A3 we noticed that a small hydrophobic patch was formed under CETP already in ∼20 ns, and the patch slowly grew in size and number as in ∼80–100 ns there were two patches close to one another (data not shown). The fact that also 100 ns atomistic simulations show the hydrophobic patch formation confirms that the CETP-lipoprotein interaction is strong specifically under the concave surface and promotes the formation of a path between the droplet core and CETP.

The hydrophobic patch formation exposes the hydrophobic parts of the lipids to the concave surface where the hydrophobic tunnel openings are located. However, hardly any of the CEs were in contact with CETP, as can be seen from Figure 4. Thus, we reduced the number of POPCs from 180 to 90 (CG3-90POPC) and simulated the system again for 2 µs in order to see if the lower surface pressure of a lipid droplet would promote the solubility of CEs to the surface lipid monolayer and the interaction between CETP and CEs. Indeed, the contacts between core CEs and CETP increased. Clearly, the concave surface of CETP has some affinity for CEs, over random thermal fluctuations, as the hydrophobic patch under CETP guides core CEs to the concave surface. However, the surface pressure must be low enough for CEs to localize to the surface monolayer and CETP to bind to the surface. This implies that the ratio of surface and core lipids (surface pressure) and the formation of the small hydrophobic patch under CETP are important factors modulating the core lipid transfer activity of CETP.

### Structure fluctuations show that the hinge region of helix X is highly mobile

**Figure 5:**
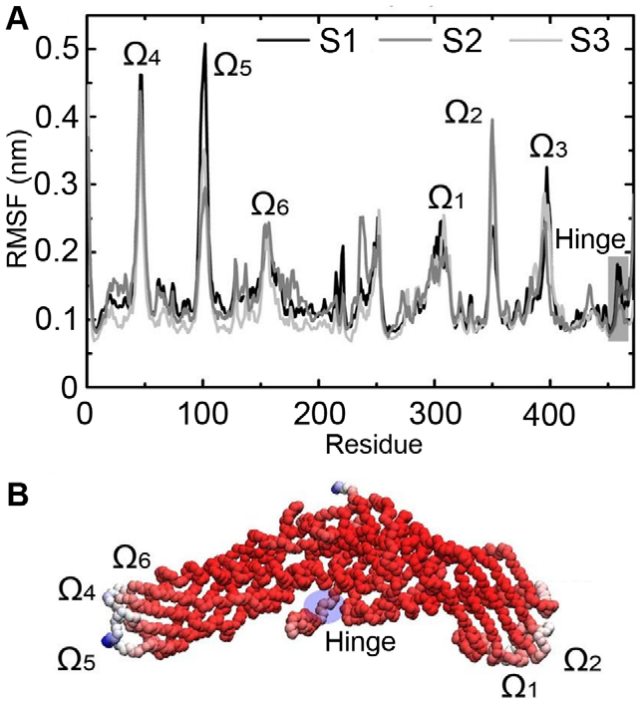
Dynamical properties of CETP. A) Root mean square fluctuations for atomistic droplet simulations. Loop regions are marked with omegas and the hinge region of helix X has been slightly darkened. B) Residual B-factors mapped to the backbone structure of CETP. Red color indicates the most rigid structures, whereas white and blue indicate the most flexible structural regions. The hinge region of helix X is marked with a transparent blue sphere.

Root mean square fluctuations (RMSFs) of the protein backbone were analyzed after 40 ns of atomistic lipid droplet simulations to find the regions, which wobble the most after CETP attached to the surface of the lipid droplet (Figure 5). This was done as follows. First, the RMSF of backbone atoms was computed by fitting the atomic positions to the reference structure (average structure of CETP after its binding to the lipid droplet surface) and then calculating the average distance deviation from the reference structure. The RMSFs of individual backbone atoms were then averaged per residue to determine the residual RMSF profile. Similar results were observed in all droplet simulations. The N- and C-terminal ends and loop regions (marked by omegas) of CETP showed high fluctuations, as expected. We also found that in the helix X region (residues 460–476) the conformational fluctuations peaked near the residue 462. This region has previously been proposed to be a potential hinge region of helix X with elevated B-factors [7]. In addition, it was found that the flaps Ω_1_ and Ω_2_ resulted in high fluctuations to the RMSF profile as was also proposed based on the B-factors of the X-ray structure of CETP [7]. In addition to the suggested high fluctuations, we found that another five regions of CETP were also highly fluctuating in each simulation. These regions were Ω_3_ (residues 380–400), Ω_4_ (residues 40–50), Ω_5_ (residues 90–110), Ω_6_ (residues 150–170), and Ω_7_ (residues 230–260). All regions are found in the loops, and hence high fluctuations can be expected. Previously it has been speculated that the hinge region could promote the needed flexibility to helix X that is important in the lipid exchange process [5]. To study further the role of helix X in lipid exchange, we did two additional atomistic simulations to probe its role in the lipid exchange process, see below.

### Helix X regulates the accessibility of cholesteryl esters inside CETP

**Figure 6:**
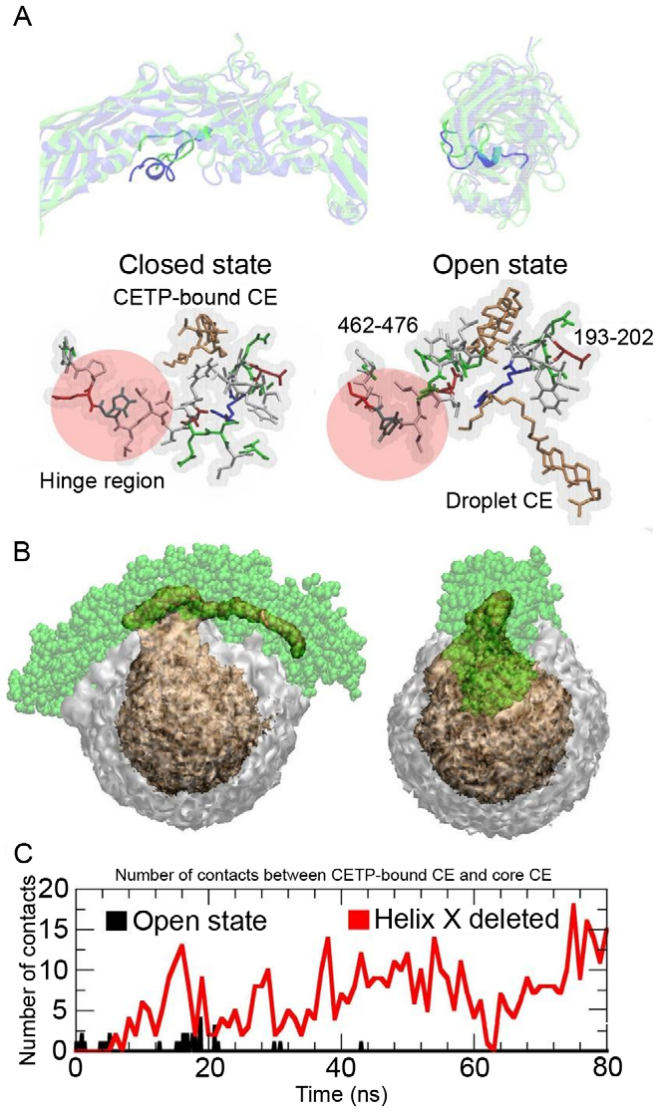
Hypothesis for the initial event of helix X assisted core lipid exchange. A) Two RMSD-fitted snapshots from the simulation A3-90POPC showing the rearrangement of helix X (darkened colour). The green conformation is for the open state and the blue one for the closed state. A more detailed structure of helix X and the role of the hinge region during the conformational change (red and transparent region) are shown in the lower snapshots. CEs are shown as orange sticks. The residues 462–476 and 193–202 of CETP have been rendered using sticks, and coloring is based on the polarity of residues. B) Spatial number density of POPCs (grey and transparent) and CEs (orange) during the simulation A3-90POPC. Core CEs diffuse into the hydrophobic tunnel of CETP (green spheres) without helix X. C) Number of contacts between core CEs and interior CE-473 when helix X is in the open state (black) or completely removed (red).

Earlier point and deletion mutations suggest that helix X is important in the transfer of core lipids, while it is not needed in phospholipid transfer [12]. Since we found that the hydrophobic patch was formed under the concave surface of CETP in both CG and atomistic simulations, we asked if the fully formed hydrophobic patch could induce changes to the conformation of helix X. To test this hypothesis, we did one additional atomistic simulation with 90 POPCs (that is, starting from the system A3-90POPC) where we expanded the hydrophobic patch under the concave structure by removing POPCs near the two tunnel openings of CETP, so that helix X was only able to interact with the hydrophobic parts of POPCs and CEs. Here, it is worth to mention that atomistic simulations are the only method of choice for this purpose, since this kind of conformational change can not take place in our CG simulations, where we used the elastic network model to keep the secondary structure of CETP stable [14]. We found that the conformation of helix X rearranged and became buried inside the hydrophobic cavity of CETP, where it interacted with CETP-bound CE (Figure 6). This conformational change generated a hydrophobic pathway from the droplet surface to the tunnel, increasing the accessibility of core CEs to the hydrophobic tunnel of CETP.

To further assess the regulatory role of helix X, we created a deletion mutant of CETP, in which the residues 462–476 (helix X) were removed from the structure, and we simulated this structure for 80 ns. Deletion mutation simulation revealed that three CEs readily diffused into CETP when helix X was completely removed from the structure (see the spatial density maps and the number of contacts plot in Figures 6B and 6C). This provides further support for the view that helix X acts as a lid at the C-terminal tunnel opening, and that its conformation regulates the accessibility of CEs to the hydrophobic tunnel.

## Discussion

Previously, the role of electrostatic interactions in the formation of isolable CETP-lipoprotein complexes was demonstrated by Pattnaik et al., who showed that (in addition to CETP-HDL complexes) CETP was able to form isolable complexes with LDL and VLDL particles when negative surface charge was increased by phospholipase A_2_ digestion or by acylation of phospholipid amino groups. They reached the conclusion that the phospholipid phosphate groups are the primary sites for the interaction of lipoproteins with CETP [8]. They also found that the formation of isolated CETP-HDL complexes was hindered by decreasing the pH, introducing positive divalent ions into the solution, or by digesting lipoproteins by phospholipase C. Moreover, Nishida et al. reported that the affinity of CETP for various lipoproteins is governed by a delicate balance of electrostatics and hydrophobic interactions [15]. The importance of electrostatic interaction in CETP binding has been shown also by several point mutation studies applied to the positively charged lysine residues of CETP [16]. Our results are in agreement with experiments, as in the present simulations most of the salt-bridges with the negatively charged phosphate groups of POPCs were formed by the Lys residues at the concave surface, when CETP fastened to the lipid surface. However, also Glu and Asp residues formed salt-bridges with the positively charged choline groups of POPCs, although the ratio of salt-bridges formed by the positively and negatively charged amino acids is approximately 1.8, indicating that mostly the positively charged amino acids contribute to the formation of CETP-lipoprotein complexes. Another important factor playing a role in the binding of CETP is Trp residues located in the flaps Ω_5_ and Ω_1_ that were found to become buried into the lipid matrix. Most likely Trp residues add more stability to the CETP-lipoprotein complexes by anchoring CETP to a lipoprotein surface. Interestingly, Desmuraux et al. made mutations to the structurally similar flap Ω_5_ region (Trp-91, Phe-92 and Phe-93) of PLTP and showed that the phospholipid transfer activity of PLTP from liposomes to HDL particles decreased up to 60% [17]. This finding together with our results suggests that the flexible flap Ω_5_ region of CETP and Trp residues therein are crucial in the binding of CETP to HDL particles, playing an important role in the CETP-mediated lipid transfer.

Penetration depth of CETP is an important factor in CETP-mediated lipid exchange, as it determines how efficiently the neutral core lipids are able to interact with CETP. Previous studies have shown that the exclusion pressure of CETP is lower than the exclusion pressure of other apolipoproteins, like apoA-I [9], [10]. Moreover, it has been argued that the weaker penetration of CETP to the emulsion particles compared to apoA-I makes the activation energy of the attachment and detachment of CETP lower, rendering the transportation process more efficient [9], [10]. Our atomistic and CG simulation results showed that CETP is not able to bury its amino acid residues deeper than to the level of the phosphate groups of POPCs. The above findings therefore imply that core lipids have to diffuse to or reside at the surface to enter CETP. Therefore, the amount of core lipids at the lipoprotein surface is an important factor modulating the activity of CETP, as has been suggested previously based on liposome studies [18], and it can be promoted by defects as is outlined below. The number of surface-located neutral lipids can be regulated by the lipid and apolipoprotein composition of lipoprotein particles.

Interestingly, we found that when CETP attaches to the surface by the aid of electrostatic interactions, the head groups of POPCs moved aside, providing access to the hydrophobic lipid region. In this manner, the two tunnel openings of the concave surface are exposed to the hydrophobic lipid matrix of the lipoprotein. The hydrophobic patch formation facilitates, by generating a defect to the surface monolayer, the diffusion of core lipids to the surface monolayer region located under CETP (Figure 6B). Thus, the localization of neutral lipids at the surface monolayer itself is not crucial to allow CETP to exchange neutral lipids between lipoproteins but the neutral lipids can enter CETP through the formation of the hydrophobic patch. Consequently, we envision that the activity of CETP could be inhibited by nonpolar drugs that are transferred into the hydrophobic tunnel of CETP through the hydrophobic core of lipoproteins. Further, we observed that the concave surface interacted directly with CEs that diffused more readily to the hydrophobic tunnel openings when the surface-core lipid ratio was decreased.

Finally, we found CEs to diffuse into the hydrophobic tunnel of CETP and interact with CETP-bound CE when the conformation of helix X was in the open state or completely removed. A previous mutational study argued that CETP is not able to transfer neutral lipids when helix X is removed from the structure [11], [12]. However, CETP is able to transfer phospholipids without helix X. Our results showed that the conformation of helix X rearranges, and helix X moves inside the hydrophobic tunnel of CETP where it can interact with CETP-bound CE. Given this, we suggest that there are two important functional properties of helix X that make the neutral lipid exchange possible. First, helix X is able to facilitate the neutral lipid exchange by opening the hydrophobic pathway from a lipoprotein surface to the hydrophobic tunnel of CETP. Second, helix X promotes the diffusion of neutral lipids from the hydrophobic tunnel to lipoproteins by filling the volume of CETP-bound neutral lipid when it diffuses out from CETP. Afterwards, another neutral lipid from the lipoprotein core or inside CETP could take the place of helix X after which the C-terminal tunnel opening closes again. We propose that helix X is needed to prevent the structure of CETP from collapsing as was registered in the simulation A1 without the CETP-bound lipids. The above reasons would explain why helix X is important in the neutral lipid exchange, but not in the exchange of phospholipids.

It is tempting to contemplate the possible roles of helix X in the inhibition of CETP. It has been reported that dalcetrapib, a novel CETP inhibitor, binds covalently to CETP by forming a disulphide bond with Cys-13, which is located inside the hydrophobic tunnel of CETP [19]. In addition, it has been suggested that the disulphide bond formation is a necessary requirement for the dalcetrapib-mediated CETP inhibition. However, that is not the case with torcetrapib or anacetrapib (another novel CETP inhibitors), both of which bind reversibly to CETP [19]. Yet, all inhibitors stabilize HDL-CETP complexes, which has been found to be the second major inhibitory mechanism of the neutral lipid transfer exerted by the synthetic CETP inhibitors [19]. Based on our simulations, we can hypothesize that helix X is locked to the open state when inhibitors are bound to CETP. The driving force for this could be the small size of an inhibitor that enforces helix X to be located inside the hydrophobic tunnel and, thus, prevents collapse of the lipid pocket. Another reason could be the more favorable interaction between helix X and the inhibitor inside the tunnel, which could conceivably force the conformation of helix X to the open state, or change the conformation distribution to favor the open state. Consequently, the detachment of CETP from the surface of HDL would be hindered since the helix X is not able to shield the hydrophobic tunnel opening of the lipid pocket when CETP is completely in the aqueous phase. In addition, the open state could prevent the binding of phospholipids to the C-terminal tunnel opening, which, based on the X-ray structure of CETP, is known to be occupied by phospholipids when CETP is not attached to a lipoprotein surface. A reduced ability of CETP to bind and transport phospholipids could further stabilize the HDL-CETP complex.

In summary, we have provided a detailed atomistic picture regarding the initial steps in the lipid exchange mechanism of CETP and, furthermore, we have offered a plausible mechanism for the exchange of neutral lipids mediated by CETP. Overall, our work paves the way for additional future studies to elucidate interactions of the available promising CETP inhibiting drugs, such as anacetrapib and dalcetrapib, with CETP and CETP-lipoprotein complexes. Our findings for the factors that affect the lipid exchange process can also be exploited in the design of novel molecular agents capable of inhibiting the activity of CETP, one possible strategy being the design of nonpolar drugs which can be transferred into the hydrophobic tunnel of CETP. Together with recent simulation models for both HDL and LDL [20], these ideas are a reasonable goal already at present.

## Materials and Methods

### System setup

The coordinate file of CETP in the PDB format with an accession code 2OBD was acquired from the RCSB Protein Data Bank. In addition to the protein, the structure provides information of the lipids carried by CETP: there are two CEs located inside the long hydrophobic tunnel of CETP, and two dioleoylphosphatidylcholine (DOPC) lipids that cover the two endings of the hydrophobic tunnel. The charge state of CETP was chosen to represent the physiological pH that is 7.4. A detailed explanation of the protein structure is given elsewhere [7].

For atomic-scale simulations, three different setups were constructed by combining pre-equilibrated lipid droplets consisting of 180 palmitoyloleoyl-PC (POPC) and 35 CE molecules [21]. In each system, CETP was placed approximately at a distance of 1 nm from the surface of the lipid droplet (Figure 2). In the first simulated system (A1), the two DOPCs and CEs were removed from CETP. In the second simulation (A2), both DOPCs and CEs were included, while in the third simulation (A3) only CEs were kept inside CETP. We also simulated CETP with a planar trilayer system composed of 512 POPCs and 796 CEs (A4). The droplet systems were solvated with ∼180,000 water molecules at a salt concentration of 0.2 M including counter ions, while the trilayer system included ∼50,000 water molecules. Altogether, the systems included ∼500,000 atoms. Finally, three additional atomistic systems were constructed to characterize the role of helix X in lipid exchange in more detail (see text). First, we studied the effect of the hydrophobic patch on the structure of helix X by removing half of the POPCs from A3 at 100 ns (A3-90POPC). Second, we also considered CETP through its helix X deletion mutant to probe the regulatory role of the helix. Third, we used A3-90POPC as a basis and removed some of the surface lipids to model the complete formation of a hydrophobic patch under the concave surface of CETP. The context of these simulations to the studied processes will become clear in the discussion below.

In addition to atomistic simulations, we carried out four coarse-grained (CG) simulations. First, the system A3 was directly coarse grained (in the text, we refer to this simulation as CG3) by using a script that is available at the homepage of the Martini force field. Second, 90 POPCs were removed from CG3, ending up in the system denoted as CG3-90POPC. The systems CG3 and CG3-90POPC were simulated under standard Martini model (see below) conditions with regard to electrostatics (using truncation of electrostatic interactions) and the water model that is non-polarizable. To clarify the influence of long-range interactions and the water model, we simulated two additional systems. That is, in the third case we focused on the system CG3 which was simulated with full electrostatics using the particle mesh Ewald (PME) method [22]. Finally, in the fourth coarse-grained model, we simulated the system CG3 using both PME and the polarizable Martini water model [13].

### Simulation parameters and force field

The GROMACS simulation package with version 4.0 was used in the simulations [23]. In atomistic studies, we used the Nose-Hoover thermostat [24], [25] with a coupling constant of 1.0 ps to set the temperature to 330 K in which the particle core is certainly in liquid state. The pressure was set to 1 bar using the Parrinello-Rahman barostat [26] with isotropic pressure coupling and a coupling constant of 0.1 ps. The van der Waals interactions were chosen to have a cutoff at 1.0 nm. Electrostatic interactions were evaluated by the particle mesh Ewald technique with a real space cut-off of 1.0 nm [22]. Water molecules were described using the SPC water model. All non-water bonds were constrained using the LINCS algorithm [27] and the SETTLE algorithm [28] was used to constrain water molecules, allowing the use of a time step of 2 fs in the integration of equations of motion. Berger parameters [29] were used for lipids, while the GROMOS53A6 force-field [30] was employed for the protein. Combination rules were introduced for the interactions between lipids and the protein. The four leading atomistic systems (A1–A4) were simulated for 100 ns, and the last two ones that focused on helix X for 80 ns. The total simulation time of atomistic simulations was 0.56 µs.

CG simulations were also carried out with GROMACS, using the Martini force field with an extension to proteins [31], [32]. The ElNeDyn elastic network model was used to keep the structure of CETP stable [14]. The Berendsen thermostat and barostat were used with time constants of 1.0 ps. Temperature was set to 320 K and isotropic pressure coupling was used with pressure set to 1 bar. Cut-off distance for electrostatic interactions was set to 1.2 nm. For Lennard-Jones interactions we used a cut-off of 1.2 nm, and Lennard-Jones interactions were shifted to zero from 0.9 nm. Time step was 25 fs. The simulation time of each CG system was beyond 2 µs, and the times reported here are given in units of the effective Martini time. All rendered figures were done by VMD [33].

## Supporting Information

Figure S1 Spatial density maps for one of the atomistic systems and for two additional coarse-grained models.
(TIF)

Figure S2 Number of intrinsic contacts of CETP as a function of time.
(TIF)

Text S1 Description of additional data for Figure S1.
(PDF)

Text S2 Description of additional data for Figure S2.
(PDF)
