# Supplementary figures and images for: Lipid Exchange Mechanism of the Cholesteryl Ester Transfer Protein Clarified by Atomistic and Coarse-grained Simulations
Source: PLoS Comput Biol. 2012 Jan 12;8(1):e1002299. doi: 10.1371/journal.pcbi.1002299 (PMC3257282; doi:10.1371/journal.pcbi.1002299)

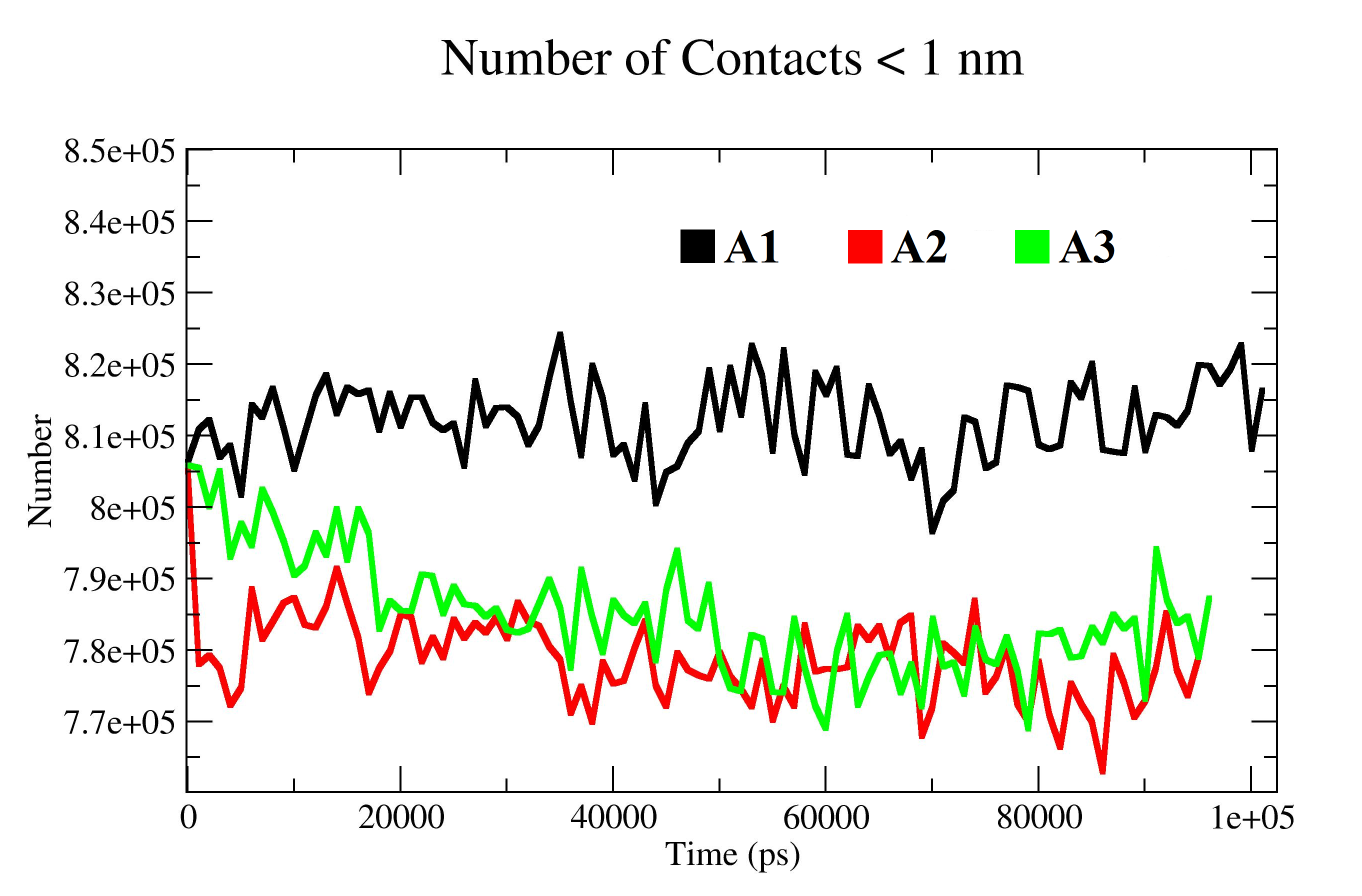

Supplement: Figure S2 — Number of intrinsic contacts of CETP as a function of time. (TIF) [file pcbi.1002299.s002.tif]
