# Supplementary material for: Lipid Exchange Mechanism of the Cholesteryl Ester Transfer Protein Clarified by Atomistic and Coarse-grained Simulations
Source: PLoS Comput Biol. 2012 Jan 12;8(1):e1002299. doi: 10.1371/journal.pcbi.1002299 (PMC3257282; doi:10.1371/journal.pcbi.1002299)
Supplement: Text S1 — Description of additional data for Figure S1. (PDF) [file pcbi.1002299.s003.pdf]

Figure S1 depicts spatial number density maps, clarifying the formation and the size of the hydrophobic patch in the different simulations. In CG simulations with PME and standard MARTINI force field parameterization, the formation of the hydrophobic patch is more pronounced that in the case of the polarizable water model. Maps from the start (0-20 ns) and end (80-100 ns) of the atomistic simulation S3 show that the hydrophobic region under CETP has grown during the simulation.

Figure S1 Spatial density maps for atomistic S3 and two additional CG simulations showing the hydrophobic patch formed to the surface of the lipid droplet. The solid and grayish surface shows where the lipid head groups are mostly located (MARTINI beads NC3 PO4, GL1, GL2 or corresponding atoms in atomistic simulation). One or two hydrophobic patches (holes on the surface of sphere) are seen under CETP (dark phantom).
