# Supplementary material for: Lipid Exchange Mechanism of the Cholesteryl Ester Transfer Protein Clarified by Atomistic and Coarse-grained Simulations
Source: PLoS Comput Biol. 2012 Jan 12;8(1):e1002299. doi: 10.1371/journal.pcbi.1002299 (PMC3257282; doi:10.1371/journal.pcbi.1002299)
Supplement: Text S2 — Description of additional data for Figure S2. (PDF) [file pcbi.1002299.s004.pdf]

Figure S2 shows intrinsic protein contacts as a function of simulation time for simulations A1, A2, and A3, indicating that without interior lipids (A1) the volume of the hydrophobic tunnel of CETP is smaller than with CETP-bound lipids (A2 and A3).

Figure S2. Number of protein-protein contacts in simulations A1, A2, and A3.
